# Scientific Evidence for Korean Medicine and Its Integrative Medical Research

**DOI:** 10.1155/2015/967087

**Published:** 2015-12-30

**Authors:** Wansu Park, Salih Mollahaliloglu, Vitaly Linnik, Han Chae

**Affiliations:** ^1^Department of Pathology, College of Korean Medicine, Gachon University, Seongnam 13120, Republic of Korea; ^2^Department of Public Health, Faculty of Medicine, Yildirim Beyazit University, 06050 Ankara, Turkey; ^3^Social Insurance Fund of Russian Federation, Moscow 107139, Russia; ^4^Division of Longevity and Biofunctional Medicine, School of Korean Medicine, Pusan National University, Busan 50610, Republic of Korea

The Korean Medicine (KM) incorporates same clinical techniques as East Asian traditional medicine; however it also has typical characteristics such as Sasang typology, Sa-am acupuncture, Chuna therapy, pharmacopuncture, and Korean psychotherapy. Modernized KM currently utilizes cutting-edge techniques of orthodox medicine, though it is rooted on the* Donguibogam*, one of the best Korean medical classics written in 1610 and enlisted on the Memory of the World by UNESCO in 2009. In 2013, the National Health Insurance of Korea paid about 2 billion dollars for medical services provided by 18,000 KM doctors in 210 KM hospitals and 13,200 KM local clinics. The role of KM in health service is expected to expand since Korea is anticipated to be an aged society in 2018 and hyperaged society in 2028 which means increased chronic disease patients. Thus the evaluation of efficacy and safety of KM and improvement of its clinical skills have been emphasized recently.

Our special issue, which had opened for 6 months in the first half of 2015, focused on scientific evidence for KM and its integrative medical research.

An article by C. Na et al. described that Jakyak-Gamcho decoction extracted with 70% ethanol exhibits higher amounts of effective index components than that extracted with water; it may be worthwhile to investigate alternative extraction methods in terms of extraction efficiency and* in vivo* effectiveness for other herbal medicines besides Jakyak-Gamcho decoction. A study by D.-S. Hwang et al. evaluated that Kyung-Ok-Ko significantly protects against heat-induced damage to testicular function in male mice by inhibiting oxidative stress and apoptosis. B. Joh et al. described that* Morus alba* treatment of infertility, jaundice, cognitive disorder, and hyperpigmentation is found to be effective and diabetes with* Morus alba* is recognized to have clinical importance. An interesting study by S. J. Lee et al. elucidated the biopsychological mechanism underlying the Sasang typology (a traditional Korean personalized medicine) using Behavioral Inhibition System (BIS)/Behavioral Activation System (BAS) scale. S. J. Lee et al. reported significant differences in BIS and BAS scores between So-Yang and So-Eum Sasang types.

K. Lee and B.-J. Lee reported the exact plant origins, efficacies, uses, components, and toxicities of Polygoni Multiflori Radix, Cynanchi Wilfordii Radix, and Cynanchi Auriculati Radix so that they can be correctly understood and used. A study by D. R. Kim et al. reported that Trigonellae semen treatment could enhance sperm function by promoting spermatogenesis and the expression of cation channel of sperm proteins in mouse testes. J.-H. Hwang et al. reported that intratracheal Chung-pae administration effectively decreases the chronic inflammation and pathological changes in a porcine pancreatic elastase- and lipopolysaccharide-induced chronic obstructive pulmonary disease mouse model. An interesting study by H.-G. Kim et al. suggested that one can find the Bonghan systems under the skin as putative acupuncture points by tracing the intraexternal Bonghan systems, from which a new KM will be born.

T.-H. Kim et al. suggested a recommendation for reporting cases of acupuncture-related infections. The recommendation includes items on patient's condition and adverse events (or complications) in detail, which are necessary to establish the causality between acupuncture and the event as well as to provide information for judging appropriateness of acupuncture practice. A study by M.-H. So and Y.-K. Choi reported that the water extracts of Scutellariae radix and Liriopis Tube significantly suppressed the increased production of nitric oxide, interleukin-6, macrophage inflammatory protein-1*α*, macrophage inflammatory protein-1ß, macrophage inflammatory protein-2, and granulocyte colony-stimulating factor as well as the increase of the intracellular free calcium in mouse macrophages induced by lipopolysaccharide. A research article by J. S. Ha et al. reported that the ethyl acetate fraction from* Actinidia arguta* containing physiological phenolics might enhance drug-induced amnesia through acetylcholinesterase inhibition and neuroprotection.

Another study by W.-M. Jung et al. demonstrated that the indications of each acupoint were primarily associated with the corresponding meridian system, using data mining methods to analyze the characteristics of the indications of each acupoint and to visualize the relationships between the acupoints and disease sites in the classic KM text* Chimgoogyeongheombang*. A study by J. Y. Park et al. suggested that* Artemisia asiatica* extract and eupatilin could cure or prevent cisplatin-induced renal toxicity without any adverse effect;* Artemisia asiatica* extract can be used in combination with cisplatin to prevent nephrotoxicity. An interesting review article by M. Park and S. Kim reported that Sa-am acupuncture, which operates with five shu points as a main aspect of treatment, has the advantage of increasing parasympathetic nerve activity and adjusting the balance of the autonomic nervous system; to maximize this effect, inserting a needle into the skin layer and providing gentle and light stimulation while considering the respiratory phase may be desirable.

A research article by K. Kim et al. reported that KM combination therapy may be beneficial for decreasing pain and improving function in lumbar spinal stenosis patients and may produce comparatively few adverse events. Another study by J. W. Suh et al. reported that the Emotional Freedom Technique, a meridian-based psychological therapy that alleviates psychologic and psychosomatic conditions by applying tapping stimulations at certain meridian acupoints, is more effective in improving anger and anxiety in the Hwabyung patients compared to the conventional meditation technique of Progressive Muscle Relaxation. An interesting study by H. G. Kim et al. suggested that those with a Taeumin type (one of four Sasang types) may tolerate psychological or oxidative stress better than those with the other types in accordance with the differences in the serum levels of stress hormones and the oxidative stress markers.

In conclusion, we expect that this special issue updates scientific evidences in KM and makes useful progress on KM integrative research.

